# Torquetenovirus Serum Load and Long-Term Outcomes in Renal Transplant Recipients

**DOI:** 10.3390/jcm9020440

**Published:** 2020-02-06

**Authors:** Edmund J. Gore, António W. Gomes-Neto, Lei Wang, Stephan J. L. Bakker, Hubert G. M. Niesters, Anoek A. E. de Joode, Erik A. M. Verschuuren, Johanna Westra, Coretta Van Leer-Buter

**Affiliations:** 1 Department of Medical Microbiology, Rijksuniversiteit Groningen, University Medical Centre Groningen, 9713GZ Groningen, The Netherlands; h.g.m.niesters@umcg.nl (H.G.M.N.); c.van.leer@umcg.nl (C.V.L.-B.); 2 Department of Internal Medicine, Rijksuniversiteit Groningen, University Medical Centre Groningen, 9713GZ Groningen, The Netherlands; a.w.gomes.neto@umcg.nl (A.W.G.-N.); s.j.l.bakker@umcg.nl (S.J.L.B.); a.a.e.joode@umcg.nl (A.A.E.d.J.); 3 Department of Rheumatology and Clinical Immunology, Rijksuniversiteit Groningen, University Medical Centre Groningen, 9713GZ Groningen, The Netherlands; l.wang@umcg.nl (L.W.); johanna.westra@umcg.nl (J.W.); 4 Department of Pulmonology, Rijksuniversiteit Groningen, University Medical Centre Groningen, 9713GZ Groningen, The Netherlands; e.a.m.verschuuren@umcg.nl

**Keywords:** torquetenovirus, immunosuppression, transplantation, immunosuppressed host, outcome, renal transplantation

## Abstract

Following transplantation, patients must take immunosuppressive medication for life. Torquetenovirus (TTV) is thought to be marker for immunosuppression, and TTV–DNA levels after organ transplantation have been investigated, showing high TTV levels, associated with increased risk of infections, and low TTV levels associated with increased risk of rejection. However, this has been investigated in studies with relatively short follow-up periods. We hypothesized that TTV levels can be used to assess long term outcomes after renal transplantation. Serum samples of 666 renal transplant recipients were tested for TTV DNA. Samples were taken at least one year after renal transplantation, when TTV levels are thought to be relatively stable. Patient data was reviewed for graft failure, all-cause mortality and death due to infectious causes. Our data indicates that high TTV levels, sampled more than one year post-transplantation, are associated with all-cause mortality with a hazard ratio (HR) of 1.12 (95% CI, 1.02–1.23) per log_10_ increase in TTV viral load, (*p* = 0.02). Additionally, high TTV levels were also associated with death due to infectious causes (HR 1.20 (95% CI 1.01–1.43), *p* = 0.04). TTV levels decrease in the years following renal transplantation, but remain elevated longer than previously thought. This study shows that TTV level may aid in predicting long-term outcomes, all-cause mortality and death due to an infectious cause in renal transplant patients sampled over one year post-transplantation.

## 1. Introduction

Immunosuppressive therapy is vital for organ transplantation medicine; in the last 20 years, antirejection treatment has improved enormously thanks to the increased availability of new antirejection drugs. All these drugs, however, lead to some degree of immunosuppression, and subsequently increased infection risk. Measuring trough levels of antirejection drugs is currently standard of care in determining the optimal dosing of these drugs, but is well recognized that these trough levels do not accurately reflect the risk of under-immunosuppression, potentially resulting in rejection, or over-immunosuppression, potentially resulting in increased infections [1,2,3]. It is, therefore, important to identify the parameters that reflect the net immune status and have predictive capacities for long-term outcomes. Torquetenovirus (TTV) is a single stranded, negative sense, non-encapsulated DNA virus; it was first discovered in 1997 by Nishizawa et al. [4,5,6] and is present in 46%–100% of healthy people [7]. The international commission on taxonomy (ICTV) recognizes 29 different genotypes, but their relative circulation has not been researched sufficiently [8]. Attempts to discover viable antibody assays have been hampered by the hypervariable nature of the viral capsid protein [9]. A few assays have been described by various groups; however, these assays have not proven to be scalable for large-scale clinical use [9,10,11]. In recent years, TTV has been studied by various research groups as a potential marker of immunosuppression following transplantation. TTV levels have been shown to increase at the start of antirejection treatment, reaching a relative plateau phase between 3 and 6 months after transplantation [12]. It is currently thought that an ideal viral loads exists for each type of organ transplantation, signifying optimal antirejection dosing. Viral loads above this ideal level have been shown to increase the chance of infections, whereas low viral loads have been shown to be associated with increased chance of rejection [13]. No formal cut-off TTV levels for optimal immunosuppression have been established, however, since these levels show great variation between different research groups, even in seemingly similar patient populations. These variations may be due to differences in circulation of TTV genotypes or due to differences in the PCR test used in these studies.

Additionally, most studies have looked at longitudinal TTV measurements relatively shortly after transplantation. The follow-up periods have also been brief, usually up to one year after solid organ transplantation (SOT). The immunosuppressed condition of these patients and the increased risk of infection nevertheless persists for a lifetime. To date, very little is known about TTV levels past the first year after transplantation, and if these TTV levels are associated with increased risk of infection or rejection in the long term. In this study, we focused on TTV measurements after the first year following renal transplantation, and hypothesized that TTV levels are associated with outcome over several years. We studied this in a cohort of 666 renal transplant recipients.

## 2. Materials and Methods

### 2.1. Study Population

This cohort study was based on a previously described set of 706 renal transplant recipients [14,15]. Included were patients (aged  ≥  18 years) who visited the outpatient clinic of the University Medical Centre Groningen (UMCG), Groningen, the Netherlands, between November 2008 and June 2011, and who had a graft that had been functioning for at least one year with no history of alcohol and/or drug addiction. Of 706 renal transplant recipients that provided written informed consent, we excluded subjects with missing biomaterial (40 cases) from further analyses, which resulted in 666 cases eligible for study. The study protocol was approved by the UMCG institutional review board (METc 2008/186); clinical trials number NCT02811835 and adhered to the Declarations of Helsinki and Istanbul.

### 2.2. TTV Viral Load Measurements

Serum samples were stored at −80 °C, until analysis. One serum sample per patient was used; this was collected at enrollment. DNA was extracted from thawed serum samples using the eMAG Nucleic Acid Extraction System (bioMerieux, Marcy, France). The Argene R-Gene TTV quantification kit (bioMerieux, Marcy, France) was used to perform qPCR on an Applied Biosystems 7500 (Thermo fisher, Waltham, MA, USA) according to the manufacturer’s instructions. Due to limited sample volumes, 100 µL, a 1 in 4 dilution using DMEM, was performed prior to sample extraction (ThermoFisher, Waltham, MA, USA). A control experiment (data not shown) showed no significant differences in the Ct values. The R gene assay is designed to detect TTV genotypes 1, 6, 7, 8, 10, 12, 15, 16, 19, 27 and 28 [16,17,18].

### 2.3. Clinical End Points

The primary endpoint of this study was all-cause mortality, death due to infectious causes and death-censored graft failure as secondary aims. Deaths due to infectious causes were defined using the previously specified list of International Classification of Diseases, Ninth Revision, codes 1–139 [19,20]. For example, a patient meeting the criteria, which is positive culture or PCR for *Pneumocystis jiroveci* infection, would be given the code 136.3 and would therefore be classified as dying due to an infection. Graft failure was defined as return to dialysis therapy or re-transplantation. The cause of graft failure was obtained from patient records and was reviewed by a blinded nephrologist. Endpoints were recorded until the end of September 2015 and there was no loss of subjects to follow-up.

### 2.4. Data Analysis

Data analyses were performed using SPSS 23.0 for Windows (SPSS Inc., Chicago, IL, USA) and R (R Foundation for Statistical Computing, Vienna, Austria). As no cut-off values for low, medium and high TTV load have been established, and to avoid bias, renal transplant recipients were stratified into three equally sized groups based on serum TTV. This created four groups, named undetectable-TTV, low-TTV, medium-TTV, and high-TTV, which were further analyzed. Differences in all-cause mortality, death due to infectious causes and graft failure between the four groups were compared using Kaplan–Meier plots and log rank tests. Data are presented as mean  ±  SD for normally distributed data, as median [interquartile range (IQR)] for non-normally distributed data, and as number (percentage) for nominal data. *t*-Tests, or one-way ANOVA tests with Tukey post-hoc tests, were performed on normally distributed data. Each group was compared with the other three groups. Kruskal–Wallis or Mann–Whitney U tests were performed on non-normally distributed data. Chi-square tests were used on categorical data. A two-sided *p*  <  0.05 was considered to indicate statistical significance in all analyses.

Prospective associations of TTV on study endpoints were explored by means of Cox regression analysis. Risk of all-cause mortality, death due to infectious causes and graft failure are presented as HR [95% confidence interval]. In these analyses, associations were adjusted in a cumulative fashion for potential confounders, including age, sex, eGFR, proteinuria (model 1), time since transplantation (model 2) and the number of immunosuppressant medications taken (model 3). Cox regression models were built in a stepwise fashion to avoid over-fitting. The proportionality of hazards for covariates was investigated by inspecting the Schoenfeld residuals. eGFR and age were included as categorical variables with equal numbers of events in each group, as eGFR and age breached the proportionality of hazards assumption as continuous variables.

The optimal cutoff values for death due to infectious causes was identified by using Youden’s Index [21] in the area under receiver operating characteristics (auROC) curve. This approach was not used for all-cause mortality as the threshold was biologically unrealistic, therefore a sensitivity of 75% was set and the threshold was calculated. Sampling by replacement was used to create 1000 bootstrapped samples of equal size from within the study population, this was then used within our multivariate Cox regression models to validate the association of the TTV DNA thresholds with risk.

To assess in further detail how TTV levels change over time, and to validate our TTV test in patients with older transplants, the patients were subdivided into two groups. The first group had had a transplant 12 to 24 months prior to analysis. The 2nd group had had transplants over 24 months prior to analysis. Cox regression analysis was also performed as previously described.

## 3. Results

### 3.1. Recipient Demographics

There was a median 4.9 [IQR 3.4–5.5] years of follow-up for our study population, with 117 (18%) of renal transplant recipients having undetectable TTV, 183 (27%) having low TTV, (median 1.52 (IQR 1.00–1.85) Log_10_copies/mL), 184 (28%) medium TTV, (median 3.00 (IQR 2.61–3.40) Log_10_copies/mL), and 182 (27%) high TTV, (median 5.52 (IQR 4.08–5.27) Log_10_copies/mL) (Table 1). Median time from transplant to TTV sampling was different for each group; undetectable TTV 7.1 (IQR 4.0–12.4) years, low TTV 6.4 (IQR 3.1–11.0) years, medium TTV 5.3 (IQR 2.2–14.3) years and high TTV 3.2 (1.0–9.0) years (*p* < 0.001).

The median prednisolone dose taken by the patients was different across the groups, 7.5 mg/day for the undetectable TTV group and 10 mg/day for low, medium, high groups (*p* = 0.02). The numbers of renal transplant recipients on calcineurin inhibitors (CNIs) were different across the groups with 36%, 47%, 61% and 75% for undetectable, low, medium and high (*p* < 0.001), respectively. There were comparable numbers of renal transplant recipients on proliferation inhibitors in each group (*p* = 0.13). Twenty-three recipients were on mono-therapy, 361 on dual-therapy and 282 were on triple-therapy post-transplant. Renal transplant recipients on mono-therapy had a lower median TTV 1.67 (IQR 0.71–2.68) Log_10_copies/mL, than recipients on dual-therapy 2.1 (IQR 0.48–3.52) Log_10_copies/mL, or on triple-therapy 3.06 (IQR 2.57–4.22) Log_10_copies/mL.

There were no differences between the groups in regard to the type of donation (living vs. post mortal, *p* = 0.45), warm ischemic time (*p* = 0.53), cold ischemic time (*p* = 0.55) or proteinuria (*p* = 0.57). The patient demographics are represented in Table 1.

### 3.2. TTV and All-Cause Mortality

Patient mortality was attributed to a variety of causes, with a total of 141 patient deaths. Fifty-eight patients (41%) died due to a cardiovascular event and 40 (28%) died due to an infection. Most infectious deaths were caused by bacteria, with 29 events, five viral illness events, two fungal infections, and finally four patients died with multiple organisms. An additional 21 patients died due to malignancy and 22 due to miscellaneous causes.

**Figure 1:**
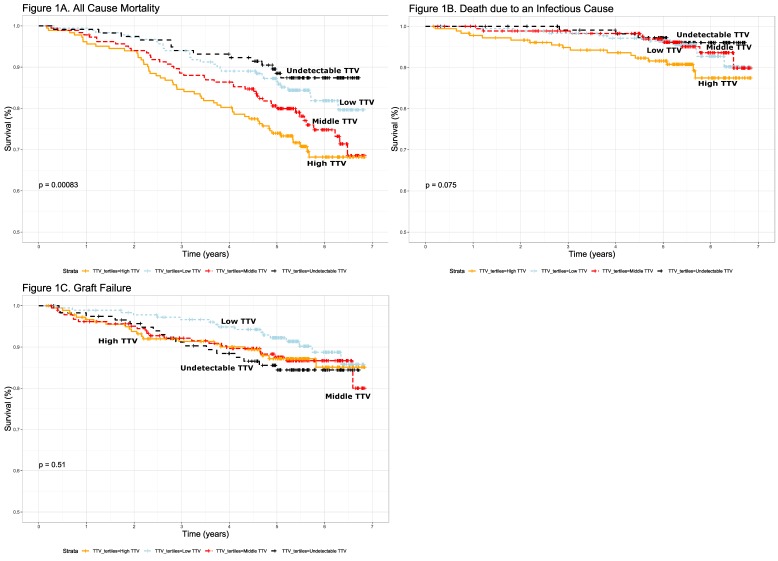
Kaplan–Meier plots showing differences in survival (**A**,**B**), and graft failure (**C**) over time between undetectable low torquetenovirus TTV (black), low TTV (light blue), medium TTV (red) and high TTV (orange), as measured in a serum sample tested at least 12 months after transplantation. Time from testing is displayed (**A**) All-cause mortality (*p* < 0.001, (**B**) Death due to infectious causes (*p* = 0.08), (**C**)) graft survival (*p* = 0.51).

**Figure 2:**
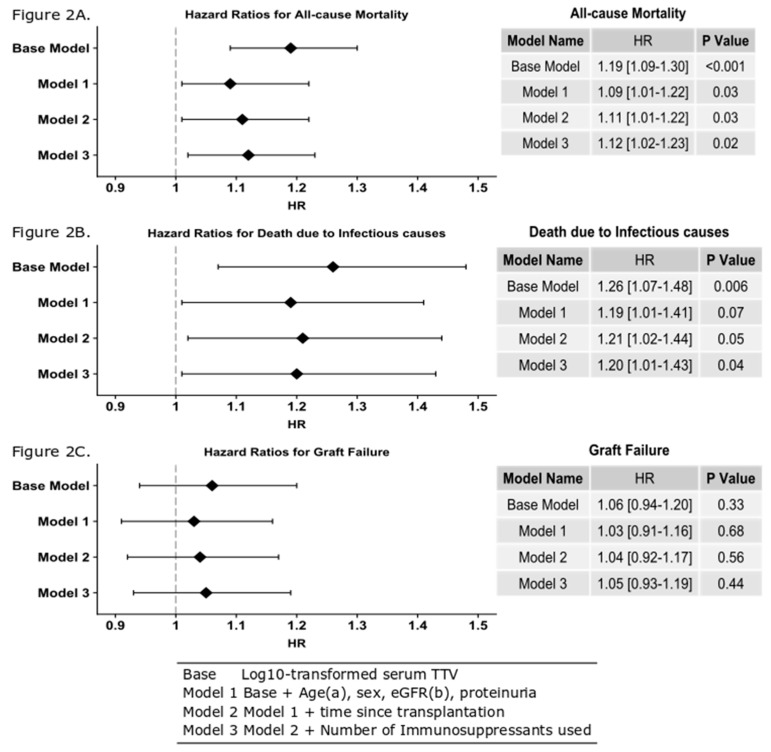
Hazard ratios calculated using Cox models and presented as a forest plot. (**A**) Log_10_ TTV is predictive of all-cause mortality after adjustment for age, gender, eGFR proteinuria, time since transplant and the number of immunosuppressant’s used. This means that for each log increase in TTV, there is a 12% increase for a patient’s risk of death (HR 1.12 (95% CI 1.0.2–1.23), *p* = 0.02). (**B**) Log_10_ TTV is predictive of death due to an infectious cause after adjustment for age, gender, eGFR proteinuria, time since transplant and the number of immunosuppressant’s used. This means that for each log increase in TTV, there is a 20% increase for a patient’s risk of death (HR 1.20 (95% CI 1.01–1.43), *p* = 0.04). (**C**) Log_10_ TTV is not predictive of graft failure after adjustment for age, gender, eGFR, proteinuria, time since transplant and the number of immunosuppressant’s used. ^a^ Categorical variable used for death due to an infectious cause. ^b^ Categorical variable used for all-cause mortality and graft failure.

We observed differences in all-cause mortality across the four categories of TTV status (log-rank test *p* < 0.001) (Figure 1A). Fourteen (12%), 30 (16%), 44 (24%), and 53 (29%) died in the undetectable group, the low group, the medium group and the high group, respectively. Time to death was shortest for the high TTV group with 5.7 (5.4–6.0) years. This compares to 6.0 (5.7–6.2) years, 6.2 (6.0–6.4) years and 6.3 (6.1–6.5) years for the medium, low and undetectable groups, respectively. As the time between transplantation and sampling was significantly shorter in the high-TTV group, it was possible that the differences in mortality and time to death were caused by disproportionally high mortality in the early years after transplantation, a period which was not observed for the patients in the low-TTV group, who were not included in the study until a median of 6.4 years after transplantation. We therefore determined the death rate within six years after transplantation and overall in the different TTV-level groups. This did not show a significant change in death rate during the follow-up period. The death rate in the first six years after transplantation was 16% in the low-TTV group and 18% overall in the follow-up period. In the high-TTV group, the mortality was 24% in the first six years after transplantation and 33% overall. It is therefore unlikely that the high all-cause mortality in the high-TTV group was attributable to the relatively early inclusion of these patients as compared to the low-TTV group. Log TTV is significantly associated with all-cause mortality in renal transplant recipients (HR 1.12 (95%CI 1.02–1.23), *p* = 0.02 per log_10_ increase in TTV), independent of potential confounders including age, gender, eGFR, time since transplantation and number of immunosuppressant medications taken (Figure 2A). Using Youden’s index, we calculated the sensitivity and specificity of a single TTV measurement, using a cut-off TTV level of 3.65 Log_10_copies/mL for identifying patients with increased chance of death; the specificity was 75% and sensitivity was 40%.

### 3.3. TTV and Death Due to a Cause

As over-immunosuppression is associated with risk of infection, we investigated the relationship between TTV levels and death due to infectious cause. In the TTV-undetectable group, four (3%) patients died from infections, whereas, 10 (6%), nine (5%) and 17 (9%) renal transplant recipients died in low group, the medium group and the high group, respectively (log-rank *p* = 0.08, Figure 1B) (Table 1). Mean time to death due to an infectious cause was not significantly different between the groups, i.e., for the undetectable group this was 6.6 (6.5–6.8) years, for the low-TTV group 6.6 (6.5–6.8) years, medium-TTV group 6.7 (6.5–6.8) years and high-TTV group 6.4 (6.2–6.6) years. Furthermore, we observed that log TTV is significantly associated with death due to infections (HR 1.20 (95% CI 1.01–1.43), *p* = 0.04), independent of potential confounders (Figure 2B). Using Youden’s index, we determined that a single TTV measurement with a level over 3.38 Log_10_copies/mL identified patients at risk of death due to infections with a sensitivity of 55%, and a specificity of 67%.

### 3.4. TTV and Graft Failure

We also observed no difference in mean graft survival across the four groups, (*p* = 0.51, Figure 1C). The numbers of patients with graft failure was not significantly different across the four groups, with 17 (15%), 17 (9%), 23 (13%) and 22 (12%) for the undetectable group, the low group, the medium group and the high group respectively (*p* = 0.57). This result is replicated when looking at the Cox model data (HR 1.01 (95% CI 0.93–1.19) *p* = 0.44, Figure 2C).

### 3.5. Time since Transplantation and TTV

**Figure 3:**
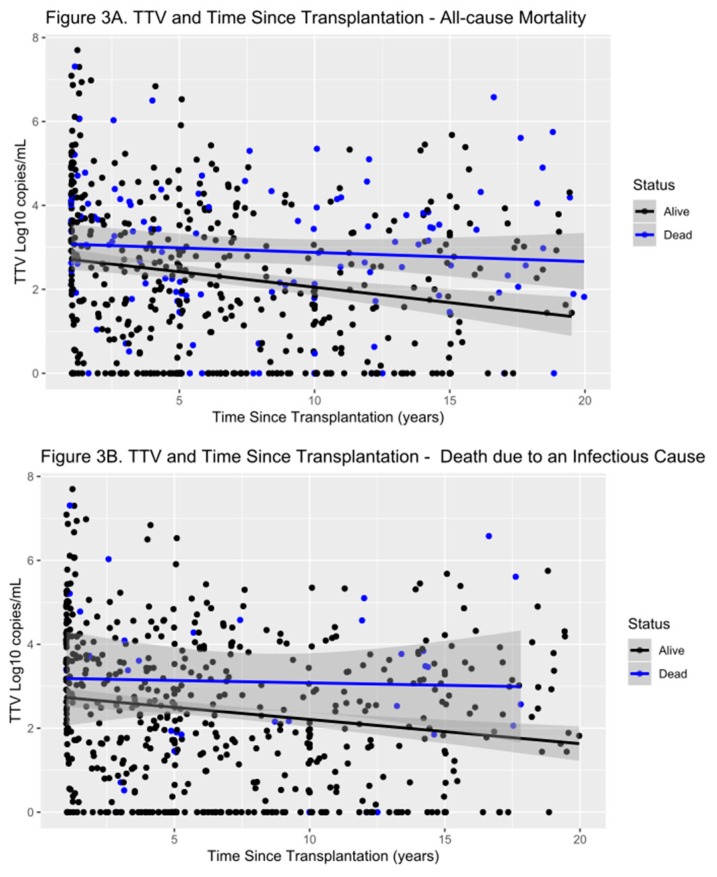
Scatter plot showing levels of TTV in our transplant patients and their outcomes in all-cause mortality (**A**), and death due to infectious causes (**B**). TTV levels are higher in the first years after transplantation than in later years. Patients with worse outcome show a trend of higher TTV levels over the entire follow-up period.

Because there is limited data on TTV levels in renal transplant recipients beyond the first year after transplantation, we divided our transplant population into two groups. One group which were sampled 12–24 months post-transplant (*n* = 164 patients) and the other over 24 months since transplant (*n* = 502 patients). This showed that TTV levels up to 24 months from transplantation were significantly higher than TTV levels in patients 2–3 years, 3–4 years, 4–5 years and over five years from transplantation (*p* < 0.05) (Figure 3).

**Figure 4:**
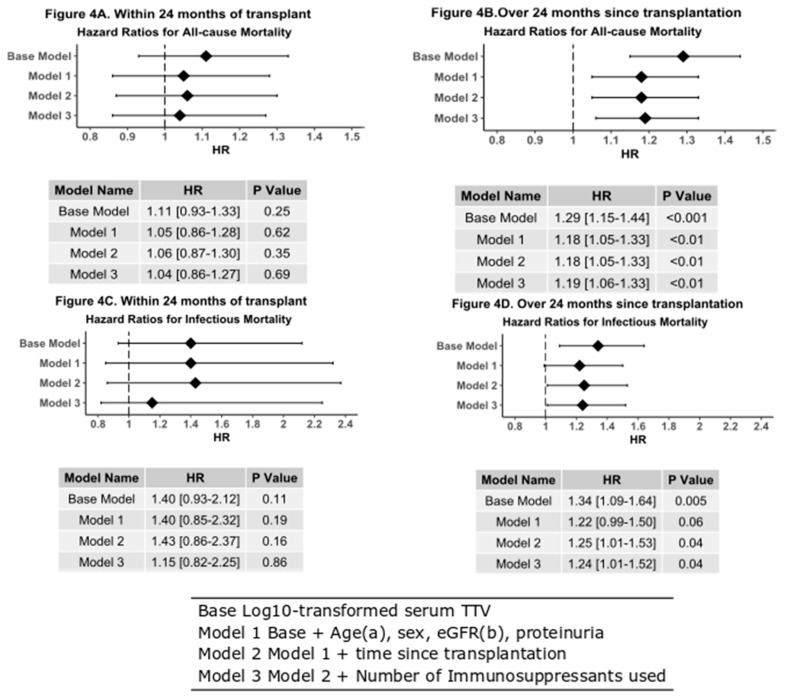
Forest plots with hazard table of Cox models after adjustment for age, gender, eGFR proteinuria, and the number of immunosuppressant’s used. (**A**) All-cause mortality in patients transplanted within 24 months of TTV analysis; this includes 31 events. Log_10_ TTV cannot be used to predict risk of death transplanted within 24 months of TTV analysis. (**B**) All-cause mortality in patients tested over 24 months after transplantation, this includes 110 events. Log_10_ TTV is highly predictive of the risk of death for patients with elevated TTV (HR 1.19 (95%CI 1.06–1.33), *p* < 0.01). (**C**) Log_10_ TTV is not predictive of death due to an infectious cause in patients tested over 24 months since transplantation (HR 1.15 (95% CI 0.82–2.25), *p* = 0.86). (**D**) Log_10_ TTV is predictive of death due to an infectious cause in patients 24 months after transplantation (HR 1.24 (95%CI 1.01–1.52), *p* = 0.04). ^a^ Categorical variable used for death due to an infectious cause. ^b^ Categorical variable used for all-cause mortality.

TTV measured within 24 months of transplantation was not associated with an increased risk of death by all causes of due to an infectious cause (Figure 4).

On the contrary, TTV measured in patients over 24 months from transplantation show that there is a significant difference in survival between patients with high, medium, low or undetectable TTV (*p* < 0.001, Figure 4). Cox modeling also shows an increased all-cause mortality when adjusting for age, gender, eGFR, and number of immunosuppressant medications taken (HR 1.18 (95% CI 0.05–1.33), *p* < 0.004). Time to death due to death due infectious causes is also shorter in the high-TTV population that are over 24 months post-transplant (*p* = 0.04, Figure 4C). This conclusion is supported by Cox analysis (HR 1.24 (1.01–1.52), *p* = 0.04). Graft failure again shows no relationship with measured TTV either within 24 months after transplantation or thereafter.

## 4. Discussion

In recent years, the use of TTV levels as a means to gauge immunosuppression has been investigated by several groups. Most of this research has used longitudinal samples taken relatively shortly after transplantation and has been aimed to predict either infection risk, due to over-immunosuppression, or rejection, due to under-immunosuppression, during the first post-transplant year. These studies have shown mixed results, with some reporting elevated TTV in patients who subsequently died of sepsis and a higher risk of CMV reactivation in patients with high TTV levels [22,23,24,25], while others showed no connection between TTV and the overall risk of infection [26]. A reason for this apparent discrepancy may be that non-specified “risk for infection” is difficult to assess, because infections are diverse and may not be observed by the transplantation center but by referring hospitals and general practitioners instead, but may also be because TTV levels are only related to certain types of infections. Because we were interested in the relationship between TTV levels and long-term outcome after renal transplantation, we focused on all-cause mortality and death due to infectious causes, as this information can be traced reliably. We found that high TTV levels are associated with both all-cause mortality and with death due to infections. The excess mortality in the high TTV group was not attributable to other factors such as age, gender, eGFR, number of immunosuppressive medications used, proteinuria and years after transplantation. Our findings suggest that TTV-levels may be predictive of much longer-term outcomes then have been investigated thus far. Other studies have shown that a relative plateau phase in TTV levels is reached in most patients, after a first period of increasing TTV levels caused by induction immunosuppression, and subsequent tapering to maintenance therapy [17]. Our study suggests that sampling during this relative plateau phase could be useful in identifying patients at risk of adverse outcomes. However, our study also suggests the relative plateau phase is relative indeed, as the TTV-levels show a decreasing trend after this first year which continues until a final stable phase develops after 24 months, and that high TTV levels are especially predictive of long-term adverse outcome if samples are taken after 24 months.

Several papers have been published looking at the role of TTV in graft failure in different types of organ transplantation [22,27,28,29,30,31]. A role for TTV measurements in predicting antibody mediated rejection in renal transplantation was suggested, with lower levels of TTV found to correlate with this type of rejection [31,32,33]. Likewise, TTV levels were also shown to be significantly lower before the diagnosis of chronic lung allograft dysfunction in lung transplant patients [31]. These results support the theory that lower levels of TTV are a risk factor for graft failure. In our study, we did not find an association between TTV and graft failure. The main reason for this may be that we looked at patients a minimum of 12 months after transplantation, past the period which represents the highest risk for acute rejection [2,34,35]. This means we likely removed several patients with a risk of rejection, reducing our power to detect rejection. Another reason for the lack of association between TTV levels and graft failure is may be that graft failure is also caused by non-immunogenic factors such as vascular damage, which TTV is unlikely to be associated with.

Although our study investigated the use of a single TTV measurement for predicting outcome after renal transplantation, and was able to show a relationship in a large group, we do not consider this method accurate enough for use in individual patients. The sensitivity and specificity of a single measurement in identifying patients with increased risk of death is so low that no real conclusions could be drawn. The cut-off TTV level which should alert clinicians to potential over immunosuppression has not become clear from this study. Our calculated cut-offs for mortality risk and infectious mortality risk are similar to the ones calculated by Fernandez-Ruiz. et al. for increased risk of infections (i.e., 3–4 Log copies/mL) [36] but are much lower than the values suggested by other authors by several orders of magnitude [22,37]. These differences stress the need for TTV assay standardization and make it difficult to draw a consensus opinion as to clinically relevant TTV viral load measurements, which would warrant action.

Nevertheless, our study results show that time since transplantation is a consideration when attempting to evaluate TTV levels as a marker for optimal immunosuppression. What may be eventually be considered a marker for either infectious risk or rejection risk may depend on when the patient is sampled.

The group of TTV-negative renal transplant recipients in our study and their collective favorable outcome after transplantation deserves attention in future research. In all studies investigating the use of TTV after transplantation, TTV-negative recipients are found. This group logically includes patients without TTV, as well as patients whose immune systems are able to suppress TTV effectively. The fact that this group has the lowest risk of death due to infections and the lowest all-cause mortality, is not surprising. However, in studies investigating TTV levels within the first year after transplantation, this group appeared to have a higher rejection risk and more graft failure. We were not able to show a connection between graft failure and TTV level. Although the set-up of our study, with a single sample taken at least one year after renal transplantation, was not inappropriate to assess acute rejection risk, the fact that there is no association between negative TTV and graft failure stresses that more investigation is needed to determine TTV level cut-offs for optimal immunosuppression after the first year post-transplantation.

When writing it was interesting to note that many studies on TTV in transplantation medicine, including this study, have come from Central and Western Europe, while the exact geographic variation of TTV has not been fully elucidated. These are also studies that, in general, have shown a correlation between TTV and various outcomes. With the advent of a minimum of four available PCR detection methods, including one commercial kit, capable of detecting various TTV genotypes with varying levels of efficiency, it would be interesting to know exactly which TTV genotype is being detected by each kit. Several authors have noted a correlation between genogroup 4 and specifically genotype 21, which has been associated with arthritis and acute respiratory disease in children [25,38]. It may be time rethink our detection strategies by using specific PCR reactions or by using sequencing more readily.

In conclusion, our data suggest a use for TTV viral load monitoring in renal transplant recipients for long term follow-up. While cut-off values remain to be determined, high TTV levels are associated with increased all-cause mortality and increased risk of death due to infections.

## Figures and Tables

**Table 1:** Recipient demographics.

	Undetectable TTV	Low	Medium	High	*p*
**Number of Patients (%)**	117 (18)	183 (27)	184 (28)	182 (27)	
Age (years)	49 ± 14 *^a^	53 ± 16	53 ± 13	55 ± 12	0.01 *
Male (%)	57 (49)	103 (56)	106 (58)	112 (62)	0.18
Weight (Kg)	77 ± 15	81 ± 16	81 ± 16	81 ± 18	0.15
BMI (Kg/m^2^)	26.0 ± 4.3	26.6 ± 4.5	27.0 ± 5.1	26.8 ± 5.0	0.33
**Renal Function**					
Serum creatine (umol/L)	132 ± 67	135 ± 63	138 ± 57	145 ± 54	0.23
eGFR (mL/1.73 m^2^)	50 ± 20 *^b^	47 ± 20 *^b^	45 ± 18	40 ± 16 *^b^	<0.001 *
Urinary protein excretion (g/24 h)	0.18 (0.00–0.27)	0.20 (0.00–0.41)	0.19 (0.00–0.47)	0.19 (0.00–0.47)	0.49
Proteinuria present, n (%)	21 (18)	42 (23)	46 (25)	40 (22)	0.57
Albuminuria (mg/24 h)	41 (8–144)	43 (11–189)	42 (12–235)	36 (9–202)	0.94
**Transplantation**					
Living Donation, n (%)	45 (39)	62 (34)	69 (38)	56 (31)	0.45
Warm Ischemic Time (minutes)	42 ± 17	43 ± 15	43 ± 16	44 ± 13	0.53
Cold Ischaemic Time (hours)	13 ± 10	14 ± 10	14 ± 11	15 ± 10	0.56
HLA I Antibodies, n (%)	9 (8)	33 (18)	26 (14)	33 (18)	0.05
HLA II Antibodies, n (%)	20 (17)	37 (20)	33 (18)	25 (14)	0.43
Transplant vintage (years)	7.1 (4.0–12.4)	6.4 (3.1–11.0)	5.3 (2.2–14.3)	3.2 (1.0–9.0)	<0.001
Acute rejection, n (%)	24 (21)	54 (30)	58 (32)	38 (21)	0.04
**Medication**					
Mono-therapy, n (%)	4 (3)	11 (6)	4 (2)	4 (2)	0.15
Dual-therapy, n (%)	82 (70)	107 (59)	94 (51)	78 (43)	0.01
Triple-therapy, n (%)	31 (27)	65 (36)	86 (47)	100 (55)	<0.001
Prednisolone dose (mg/day)	7.5 (7.5–10)	10 (7.5–10)	10 (7.5–10)	10 (7.5–10)	0.02
MTOR inhibitors, n (%)	3 (3)	9 (5)	3 (2)	3 (2)	0.17
Cyclosporin, n (%)	24 (21)	62 (34)	82 (45)	91 (50)	<0.001
Tacrolimus, n (%)	18 (15)	24 (13)	30 (16)	46 (25)	0.03
Azathioprine, n (%)	25 (21)	27 (15)	38 (21)	23 (13)	0.15
Mycophenolate, n (%)	79 (68)	126 (69)	117 (64)	120 (66)	0.94
**End Points**					
All-cause mortality	6.3 (6.1–6.5)	6.2 (6.0–6.4)	6.0 (5.7–6.2)	5.7 (5.4–6.0)	0.001
Infectious Death	6.6 (6.5–6.8)	6.6 (6.5–6.8)	6.7 (6.5–6.8)	6.4 (6.2–6.6)	0.08
Graft Failure	6.1 (5.8–6.4)	6.5 (6.3–6.6)	6.3 (6.0–6.5)	6.3 (6.0–6.5)	0.5

* a Tukey post-hoc undetectable TTV vs. TTV low, medium, high groups *p* = 0.01. * b Tukey post-hoc undetectable TTV vs. TTV low group *p* <0.001. BMI: body mass index, eGFR: estimated glomerular filtration rate, MTOR: mammalian target of Rapamycine.
